# Estrogen-related receptor β deficiency alters body composition and response to restraint stress

**DOI:** 10.1186/1472-6793-13-10

**Published:** 2013-09-22

**Authors:** Mardi S Byerly, Roy D Swanson, G William Wong, Seth Blackshaw

**Affiliations:** 1 Department of Physiology, Johns Hopkins University School of Medicine, Baltimore, MD, USA; 2 Department of Neuroscience, Johns Hopkins University School of Medicine, Baltimore, MD, USA; 3 Department of Neurology, Johns Hopkins University School of Medicine, Baltimore, MD, USA; 4 Department of Ophthalmology, Johns Hopkins University School of Medicine, Baltimore, MD, USA; 5 Center for Metabolism and Obesity Research, Johns Hopkins University School of Medicine, Baltimore, MD, USA; 6 Center for High-Throughput Biology, Johns Hopkins University School of Medicine, Baltimore, MD, USA; 7 Institute for Cell Engineering, Johns Hopkins University School of Medicine, Baltimore, MD, USA

## Abstract

**Background:**

Estrogen-related receptors (ERRs) are orphan nuclear hormone receptors expressed in metabolically active tissues and modulate numerous homeostatic processes. ERRs do not bind the ligand estrogen, but they are able to bind the estrogen response element (ERE) embedded within the ERR response elements (ERREs) to regulate transcription of genes. Previous work has demonstrated that adult mice lacking *Errβ* have altered metabolism and meal patterns. To further understand the biological role of *Errβ*, we characterized the stress response of mice deficient for one or both alleles of *Errβ*.

**Results:**

*Sox2*-*Cre*:*Errβ* mice lack *Errβ* expression in all tissues of the developing embryo. *Sox2*-*Cre*:*Errβ*^+/*lox*^ heterozygotes were obese, had increased *Npy* and *Agrp* gene expression in the arcuate nucleus of the hypothalamus, and secreted more corticosterone in response to stress. In contrast, *Sox2*-*Cre*:*Errβ*^*lox*/*lox*^ homozygotes were lean and, despite increased *Npy* and *Agrp* gene expression, did not secrete more corticosterone in response to stress. *Sox2*-*Cre*:*Errβ*^+/*lox*^ and *Sox2*-*Cre*:*Errβ*^*lox*/*lox*^ mice treated with the Errβ and Errγ agonist DY131 demonstrated increased corticotropin-releasing hormone (*Crh*) expression in the paraventricular nucleus of the hypothalamus, although corticosterone levels were not affected. *Nes*-*Cre*:*Errβ*^*lox*/*lox*^ mice, which selectively lack Errβ expression in the nervous system, also demonstrated elevated stress response during an acoustic startle response test and decreased expression of both *Crh* and corticotropin-releasing hormone receptor 2 (*Crhr2*).

**Conclusions:**

Loss of *Errβ* affects body composition, neuropeptide levels, stress hormones, and centrally-modulated startle responses of mice. These results indicate that *Errβ* alters the function of the hypothalamic-pituitary-adrenocortical axis and indicates a role for Errβ in regulating stress response.

## Background

ERRs are nuclear hormone receptors that regulate multiple homeostatic processes throughout life [1]. ERRs were initially identified on the basis of sequence homology to estrogen receptors (ERs) [2]. The homology between Errs and Ers is 36% in the ligand binding domain and 68% in the DNA binding domain. ERRs bind both ERR response elements (ERREs) and the closely related estrogen response elements (EREs) embedded within an ERRE sequence on DNA to modulate transcription of target genes [3-8]. Errs activate gene transcription by binding to DNA, either as a monomer, homodimer, or a heterodimer complex, which includes two different Err isoforms [1,6,7,9,10]. While their binding sites are similar to those of Ers, Errs do not bind estradiol and instead activate transcription in a ligand-independent manner, leading to their classification as orphan nuclear receptors. The three different *Err* genes, *α*, *β* and γ, have highly conserved ligand and DNA binding domains and thus may regulate homeostatic processes in a compensatory manner [11].

In mice, *Errβ* and *Err*γ are selectively expressed in the brain and multiple peripheral tissues [2,12-14] and share the highest degree of sequence homology [11], suggesting that they may share overlapping functions. Since Errs recognize the same response elements, they are likely to regulate overlapping subsets of target genes [11].

We have previously reported that whole-body or central nervous system-specific deletion of *Errβ* increases expression of *Err*γ and ultimately alters body composition, metabolism, meal patterns, and energy expenditure of mice [11]. Further, inhibition of *Errβ* or *Err*γ alter metabolic parameters, whole-body energy balance (e.g. body composition, food intake and neuropeptide expression), while deletion of *Errβ* reciprocally modulates expression of *Errγ* (and vice versa) suggesting that balanced expression of *Errβ* and *Err*γ is important for control of energy balance and food intake [14-18].

Alterations in glucocorticoid signaling and whole-body energy balance positively correlate with one another, with increased glucocorticoid levels resulting in increased body weight [19-21]. Errβ suppresses glucocorticoid receptor activity in neuroblastoma and kidney cells in a dose-dependent manner, suggesting that it may also regulate metabolism at least in part through modulation of the hypothalamic-pituitary-adrenal (HPA) axis [22]. The HPA axis is regulated by corticotrophin-releasing hormone (Crh) released from neurosecretory cells of the hypothalamic paraventricular nucleus. Crh stimulates release of adrenocorticotropic hormone (ACTH) from the anterior pituitary, and ACTH, in turn, triggers glucocorticoid secretion from the adrenal gland. Negative feedback from ACTH and glucocorticoid secretion ultimately modulates *Crh* expression in the paraventricular nucleus via glucocorticoid receptors [23]. Disrupting glucocorticoid feedback loops can alter whole-body energy balance (e.g. body weight). Glucocorticoid excess (Cushing’s disease) increases central fat deposition, whereas decreased body weight is associated with glucocorticoid insufficiency (Addison’s disease) [19-21]. In addition to these effects on metabolism, alterations in the HPA axis can also influence anxiety and stress, which increase Neuropeptide Y (Npy) secretion. Npy further augments obesity susceptibility by inducing food intake and contributing to leptin resistance [23-25].

Consequently, we propose that *Errβ* modulates stress responses. Since Errβ suppresses glucocorticoid receptor activity [22], we hypothesized that the HPA axis may be altered in mice that carry heterozygous or homozygous loss of function mutations of *Errβ* in all somatic tissues [14,26,27]. The effects of *Errβ* deficiency on body weight, body composition, neuropeptide levels, stress hormones, and stress responses were examined in *Sox2*-*Cre*:*Errβ*^+/*lox*^ and *Sox2*-*Cre*:*Errβ*^*lox*/*lox*^ mice, in which *Errβ* expression is disrupted in all somatic tissues. These results indicate that *Errβ* modulates stress responses, at least in part through central mechanisms.

## Results

### *Errβ* gene dosage alters body weight and body composition

*Sox2*-*Cre*:*Errβ*^+/*lox*^ heterozygous mice express one allele of *Errβ*, resulting in higher levels of *Errβ* expression relative to *Sox2*-*Cre*:*Errβ*^*lox*/*lox*^ homozygous mice. Alterations in energy balance are observed in mice deficient for *Errβ* in all embryonic tissues (*Sox2*-*Cre*:*Errβ*^*lox*/*lox*^) [14]. Because *Errβ* is proposed to modulate energy balance in a dose-dependent manner, we characterized *Sox2*-*Cre*:*Errβ*^*lox*/*lox*^ and *Sox2*-*Cre*:*Errβ*^+/*lox*^ mice to determine whether gene dosage altered development of body weight and body composition. We previously showed that *Sox2*-*Cre*:*Errβ*^*lox*/*lox*^ mice have decreased body weight and fat mass by nine months of age [8]. Body weight and body composition (fat mass and lean mass) were measured in *Sox2*-*Cre*:*Errβ*^*lox*/*lox*^, *Sox2*-*Cre*:*Errβ*^+/*lox*^, and WT mice at three weeks and at nine months of age (Table 1). By three weeks, body composition differences began to emerge between the genotypes: *Sox2*-*Cre*: *Errβ*^+/*lox*^ mice significantly increased fat mass (fat mass: *F*_1,8_ = 9.32, *P* = 0.05), while *Sox2*-*Cre*:*Errβ*^*lox*/*lox*^ mice trended toward decreased fat mass (fat mass: *F*_1,10_ = 4.95, *P* = 0.05) compared to WT mice. There was no difference in body weight among the genotypes at three weeks, implying that alterations in body composition arise prior to weight changes in *Errβ*-deficient mice.

**Table 1:** **Body weight and body composition**, **physical activity and meal patterns of wild type** (**WT**), ***Sox2***-***Cre***:***Errβ***^+/***lox***^, **and *Sox2***-***Cre***:***Errβ***^***lox***/***lox***^**mice**

**Genotype**	**Age**	**Body composition**				**Meal patterns**		
		**Body weight**	**Fat mass**	**Lean mass**	**Activity**	**Pellets**	**Satiety ratio**	**IMI**
		**(grams)**	**(grams)**	**(grams)**	**(beam breaks)**	**(number)**	**(IMI/meal size)**	**(minutes)**
WT	3 weeks	13.1 ± 0.5	2.09 ± 0.09	10.06 ± 0.41				
*Sox2*-*Cre*:*Errβ*^+/*lox*^	3 weeks	14.0 ± 0.7	2.58 ± 0.16*	10.56 ± 0.62*				
*Sox2*-*Cre*:*Errβ*^*lox*/*lox*^	3 weeks	12.9 ± 0.8	1.64 ± 0.17	10.51 ± 0.61				
WT	9 months	36.5 ± 0.9	12.19 ± 0.65	23.09 ± 0.46	67207 ± 8601	128 ± 15	8.3 ± 0.9	107 ± 17
*Sox2*-*Cre*:*Errβ*^+/*lox*^	9 months	46.1 ± 3.0*	21.43 ± 1.84*	24.00 ± 0.63	93599 ± 9879	238 ± 35* 5.6 ± 0.7^#^	102 ± 10	
*Sox2*-*Cre*:*Errβ*^*lox*/*lox*^	9 months	28.4 ± 1.3*	5.69 ± 0.74*	21.64 ± 0.40*	133741 ± 20533*	260 ± 57*	4.1 ± 0.7*	67 ± 11*

*P < 0.05 relative to WT.

^#^*P* = 0.05 relative to WT.

Activity is a measurement for the number of beam break, which represents horizontal physical activity that is parallel to the ground.

Meal patterns for *Sox2*-*Cre*:*Errβ*^*lox*/*lox*^ mice are adapted from [14].

At nine months of age, *Sox2*-*Cre*:*Errβ*^+/*lox*^ mice had increased fat mass and no change in lean mass relative to WT mice (fat mass: *F*_1,9_ = 35.90, *P* = 0.002). However, *Sox2*-*Cre*:*Errβ*^*lox*/*lox*^ mice demonstrated the opposite trend in body composition, with decreases in both fat and lean mass (fat free mass) relative to WT mice (fat mass: *F*_1,10_ = 46.53, *P* < =0.0001; lean mass: *F*_1,10_ = 6.21, *P* = 0.03). Accordingly, body weight increased in *Sox2*-*Cre*:*Errβ*^+/*lox*^ mice (*F*_1,9_ = 32.31, *P* = < 0.000001) and decreased in *Sox2*-*Cre*:*Errβ*^*lox*/*lox*^ mice (*F*_1,10_ = 32.57, *P* = 0.0004) relative to WT mice. Given these differences, the *Sox2*-*Cre*:*Errβ*^+/*lox*^ mice surprisingly had a similar macrostructure of food intake as the *Sox2*-*Cre*:*Errβ*^*lox*/*lox*^[14], relative to WT mice. Specifically, after consuming a meal, the duration of time that the mouse was satiated was decreased (satiety ratio), the total number of pellets consumed was increased, and the duration of time between meals (intermeal interval, IMI) was not changed for *Sox2*-*Cre*:*Errβ*^+/*lox*^ mice, but IMI was decreased for *Sox2*-*Cre*:*Errβ*^*lox*/*lox*^ mice (Table 1). The difference in IMI between the genotypes may be a compensatory change due to peripheral signals modulated by the increases in both body weight and fat mass observed in the *Sox2*-*Cre*:*Errβ*^+/*lox*^ mice.

### Hypothalamic neuropeptide expression in *Errβ* mutant mice

**Figure 1:**
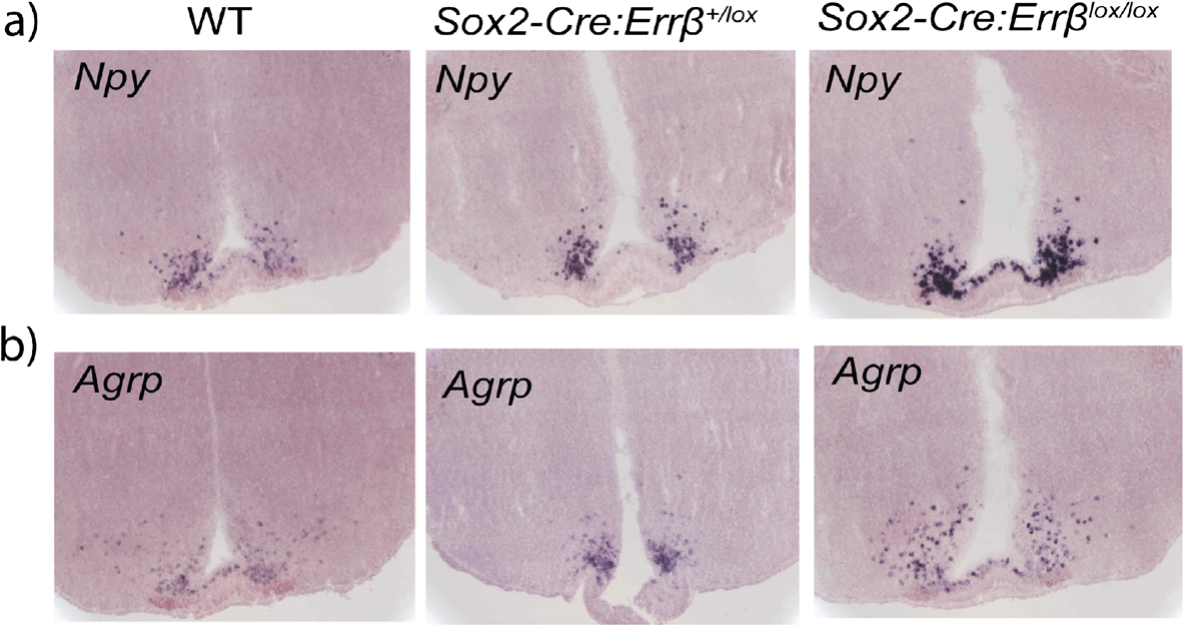
**Hypothalamic neuropeptide expression in wild-type (WT), *Sox2*-*Cre*:*Errβ***^+/***lox***^**, and *Sox2*-*Cre*:*Errβ***^***lox***/***lox***^**mouse brains.** Brain tissues were harvested from three-week-old WT, *Sox2*-*Cre*:*Errβ*^+/*lox*^, and *Sox2*-*Cre*:*Errβ*^*lox*/*lox*^ mice; frozen tissue sections were hybridized *in situ* with cRNA probes to **a)***Npy* and **b)***Agrp* (n = 3/genotype).

In the brain, *Errβ* is primarily expressed in the hindbrain, whereas *Errγ* is expressed in both the hindbrain and hypothalamus [14,28,29]. Nuclei of the hindbrain send primary projections to the hypothalamus (e.g., nucleus tractus solitarius to the paraventricular nucleus) and the amygdala, and activity in these nuclei can modulate hypothalamic gene expression [30-32]. Furthermore, in the absence of *Errβ*, Errγ can modulate food intake [14]. Since *Sox2*-*Cre*:*Errβ*^+/*lox*^ and *Sox2*-*Cre*:*Errβ*^*lox*/*lox*^ mice demonstrated alterations in body weight and body composition relative to WT mice, we sought to determine if hypothalamic neuropeptides known to modulate energy balance, *Npy* and *Agrp*, were differentially expressed in the brains of these mutants. Brain tissue sections of three-week-old WT, *Sox2*-*Cre*:*Errβ*^+/*lox*^, and *Sox2*-*Cre*:*Errβ*^*lox*/*lox*^ mice were hybridized with cRNA probes specific to *Npy* and *Agrp* mRNA. *Npy* (Figure 1a) and *Agrp* (Figure 1b) staining were least intense in the hypothalamus of WT brain tissues, more intense in *Sox2*-*Cre*:*Errβ*^+/*lox*^ brain tissues, and most intense in *Sox2*-*Cre*:*Errβ*^*lox*/*lox*^ brain tissues. Expression of *Npy* and *Agrp*, as determined by hypothalamic ISH staining, appears to correlate inversely with *Errβ* expression. Increased staining expression of *Npy* and *Agrp* may contribute to the increased fat mass of three-week-old *Sox2*-*Cre*:*Errβ*^+/*lox*^ mice; conversely, the high levels of *Npy* and *Agrp* in *Sox2*-*Cre*:*Errβ*^*lox*/*lox*^ mice may be a downstream response to decreased fat mass.

*Sox2*-*Cre*:*Errβ*^*lox*/*lox*^ mice show elevated activity levels due to defects in vestibular system development [14,26], which likely contribute to the body weight and body composition differences observed at nine months of age. However, three-week-old *Sox2*-*Cre*:*Errβ*^+/*lox*^ mice are not hyperactive, suggesting that activity alone does not control hypothalamic neuropeptide levels (Table 1: *Sox2*-*Cre*:*Errβ*^*lox*/*lox*^ vs. WT - *F*_1,9_ = 16.43, *P* = 0.004).

### *Errβ* gene dosage alters expression of HPA axis components

Errβ interacts with glucocorticoid receptors in neuroblastoma and kidney cells [22,33] and may also interact in the hindbrain where Errβ is expressed [14]. Since increased *Npy* expression is often associated with elevated levels of glucocorticoid release, which can influence adiposity [23-25], we hypothesized that *Errβ* deficiency may alter stress responsiveness via glucocorticoid secretion. Therefore, stress responses of WT, *Sox2*-*Cre*:*Errβ*^+/*lox*^, and *Sox2*-*Cre*:*Errβ*^*lox*/*lox*^ mice were measured by detecting alterations in HPA axis components, *Crh* expression and corticosterone.

**Figure 2:**
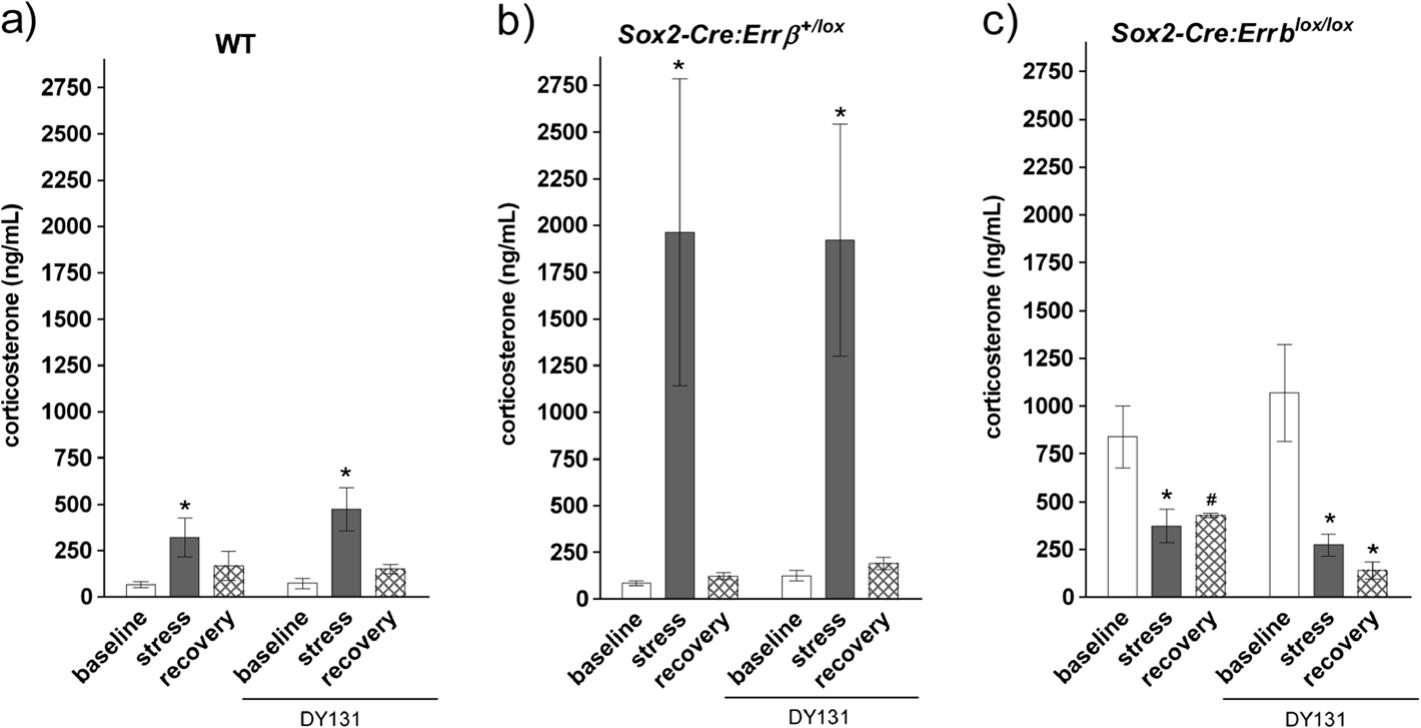
**Glucocorticoid levels of wild-type (WT), *Sox2*-*Cre*:*Errβ***^+/***lox***^, **and *Sox2***-***Cre*:*Errβ***^***lox***/***lox***^**mice after restraint stress. a)** Baseline, stress, and recovery glucocorticoid levels were measured in serum of WT mice and after treatment with Errβ/Errγ agonist DY131 using a corticosterone radioimmunoassay. **b)** Baseline, stress, and recovery glucocorticoid levels were measured in serum of *Sox2*-*Cre*:*Errβ*^+/*lox*^ mice and after treatment with Errβ/Errγ agonist DY131 using a corticosterone radioimmunoassay. **c)** Baseline, stress, and recovery glucocorticoid levels were measured in serum of *Sox2*-*Cre*:*Errβ*^*lox*/*lox*^ mice and after treatment with Errβ/Errγ agonist DY131 using a corticosterone radioimmunoassay. *P < 0.05.

To investigate the ability of Errγ to compensate for Errβ deficiency, stress responses were investigated in the presence of synthetic agonists of Errγ. Atlhough agonists specific to individual Err isoforms are not commercially available, we were able to perform these studies using DY131, a selective agonist of both Errβ and Errγ [34]. It has been previously determined that DY131 is able to readily penetrate the blood–brain barrier, as it is both hydrophobic and has a topological surface area (TPSA) less than 70 [14]. In *Sox2*-*Cre*: *Errβ*^*lox*/*lox*^ null mice, DY131 would exclusively activate Errγ and that this would result in alterations in HPA axis function (e.g. *Crh* expression or corticosterone levels). We utilized a restraint stress paradigm to measure corticosterone serum levels during baseline, stress, and recovery phases. WT mice demonstrated increased stress-induced corticosterone levels, which returned to baseline after one hour of recovery (Figure 2a) (baseline vs. stress: *F*_1,8_ = 7.82, *P* = 0.03). Similar results were measured in WT mice administered DY131 (DY131 WT, baseline vs stress: *F*_1,8_ = 6.46, *P* = 0.03; control WT; DY131 WT, stress vs recovery: *F*_1,11_ = 8.54, *P* = 0.01). *Sox2*-*Cre*:*Errβ*^+/lox^ mice exhibited markedly elevated corticosterone levels during stress, which may arise from altered negative feedback mechanisms that modulate corticosterone secretion (e.g. enhanced Crh secretion from the brain). *Sox2*-*Cre*:*Errβ*^+/lox^ mice exhibit normal recovery to baseline one hour after the stress test (Figure 2b – control *Sox2*-*Cre*:*Errβ*^+/*lox*^, baseline vs stress: *F*_1,14_ = 8.62, *P* = 0.01). Administration of DY131 yielded similar results (DY131 *Sox2*-*Cre*:*Errβ*^+/*lox*^, baseline vs stress: *F*_1,14_ = 7.02, *P* = 0.02; control *Sox2*-*Cre*:*Errβ*^+/*lox*^, stress vs recovery: *F*_1,14_ = 7.14, *P* = 0.02; DY131 *Sox2*-*Cre*:*Errβ*^+/*lox*^, stress vs recovery: *F*_1,14_ = 8.83, *P* = 0.01).

**Figure 3:**
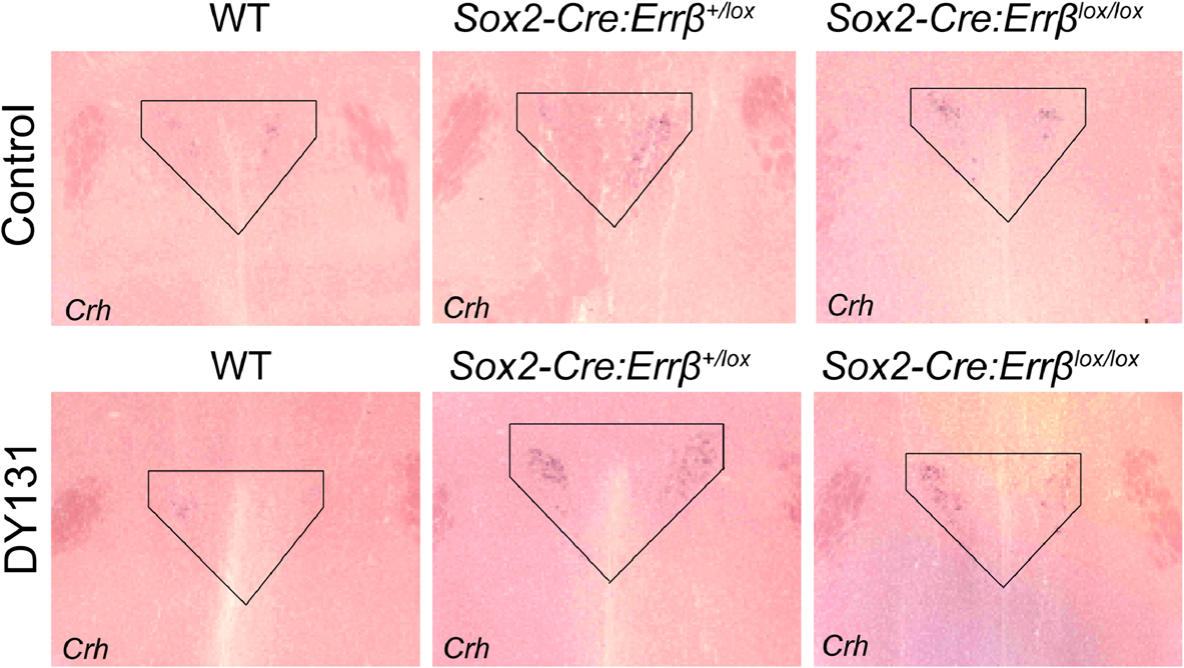
***Crh *expression of wild-type (WT), *Sox2*-*Cre*:*Errβ***^+/***lox***^**, and *Sox2*-*Cre*:*Errβ***^***lox***/***lox***^**mice.** Brain tissue of WT, *Sox2*-*Cre*:*Errβ*^+/*lox*^, and *Sox2*-*Cre*:*Errβ*^*lox*/*lox*^ mice injected with saline (top) or Errβ/Errγ agonist DY131 (bottom) were stained for *Crh* by ISH (n = 2/genotype).

In contrast, *Sox2*-*Cre*:*Errβ*^*lox*/*lox*^ mice had elevated baseline corticosterone levels but exhibited no increase with stress (Figure 2c – control *Sox2*-*Cre*:*Errβ*^*lox*/*lox*^, baseline vs stress: *F*_1,8_ = 10.86, *P* = 0.02; DY131 *Sox2*-*Cre*:*Errβ*^*lox*/*lox*^, baseline vs stress: *F*_1,8_ = 15.14, *P* = 0.01; control *Sox2*-*Cre*:*Errβ*^*lox*/*lox*^; DY131 *Sox2*-*Cre*:*Errβ*^*lox*/*lox*^, baseline vs recovery: *F*_1,8_ = 21.81, *P* = 0.01), suggesting that *Errβ*:*Sox2*-*Cre*^*lox*/*lox*^ mice are unable to increase corticosterone levels in response to restraint stress. In fact, expression of *Crh*, as determined by ISH staining, was increased in the *Sox2*-*Cre*:*Errβ*^*lox*/*lox*^ mice under baseline conditions, a modest increase in ISH staining was also seen in the *Sox2*-*Cre*:*Errβ*^+/*lox*^ mice, with DY131 further increasing the ISH staining for *Crh*. This data suggests that Errγ may modulate expression of *Crh* in a manner dependent on the level of *Errβ* expression (Figure 3).

### Neural progenitor-specific deletion of *Errβ* alters acoustic startle response

*Sox2*-*Cre*:*Errβ*^+/*lox*^ and *Sox2*-*Cre*:*Errβ*^*lox*/*lox*^ mice demonstrate differences in HPA activation, which may arise from central and/or peripheral mechanisms. In the central nervous system, *Errβ* expression is restricted to the hindbrain. *Nes*-*Cre*:*Errβ*^*lox*/*lox*^ mice lack *Errβ* in neural progenitor cells, effectively resulting in selective loss of Errβ expression in the hindbrain [14]. Therefore, we investigated the central role of *Errβ* in modulating stress responses in *Nes*-*Cre*:*Errβ*^*lox*/*lox*^ and WT mice using an acoustic startle test. The neuroanatomical and neurochemical basis of the acoustic startle response has been well mapped and involves neurons found in the amygdala, dorsomedial hypothalamus, and brainstem [35-39]. The amygdala elicits behavioral stress responses associated with the acoustic startle response and expresses the neuromodulators Crh and Npy [36,40]. *Nes*-*Cre*:*Errβ*^*lox*/*lox*^ mice have decreased *Npy* expression in the hindbrain [14], which may modify neural circuitry activated by physical and psychological stress and, more specifically, the acoustic startle response.

**Figure 4:**
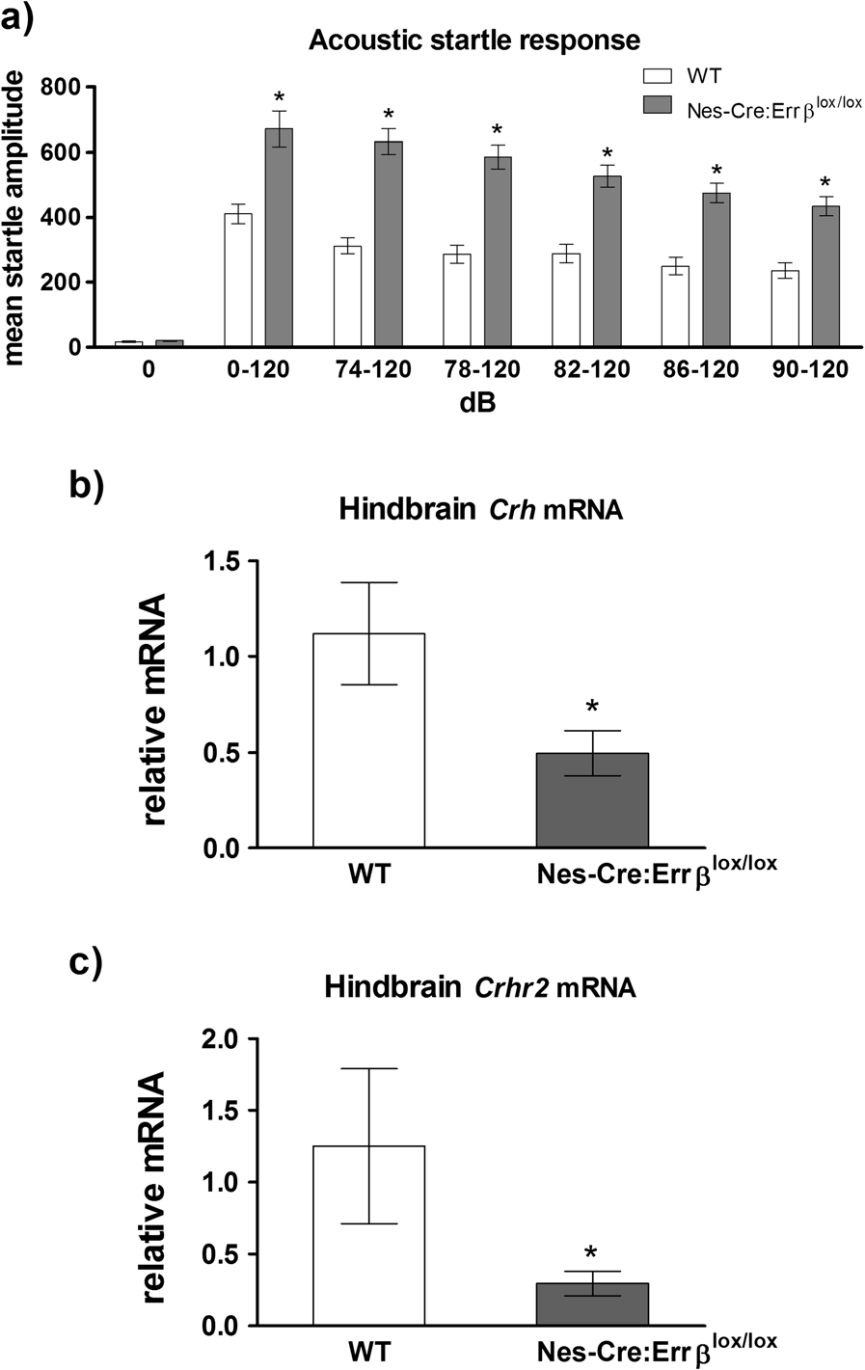
**Acoustic startle response, pre-pulse inhibition (PPI) and *Crhr2 *expression for wild-type (WT) and *Nes*-*Cre*:*Errβ***^***lox***/***lox***^**mice. a)** The force elicited by the acoustic startle response was measured from WT and *Nes*-*Cre*:*Errβ*^*lox*/*lox*^ mice after pre-pulse inhibition over five different acoustic intensities (74, 78, 82, 86, and 90 dB). **b)***Crh* and **c)***Crhr2* levels were decreased in the hindbrain of *Nes*-*Cre*:*Errβ*^*lox*/*lox*^ mice, relative to WT. Data shown are mean ± SEM for each group. *P < 0.05.

We measured PPI and the acoustic startle response to determine if *Nes*-*Cre*:*Errβ*^*lox*/*lox*^ mice had alterations in stress responses that arise from dysfunction of the inhibitory hindbrain circuit associated with PPI or the excitatory circuit associated with the acoustic startle response [41]. The acoustic startle response was measured after delivery of a prepulse intensity signal (0, 74, 78, 82, 86, or 90 dB) followed by the lead interval to a strong auditory stimulus. We observed a greater startle response in *Nes*-*Cre*:*Errβ*^*lox*/*lox*^ mice (n = 8, db120; 1081.5 ±150) compared to WT mice (n = 12, db120; 475.8 ± 27) (Figure 4a, 0db - *F*_1,20_ = 0.05, *P* = 0.81; 0-120db - *F*_1,20_ = 9.25, *P* = 0.006; 74-120db - *F*_1,20_ = 15.13, *P* = 0.001; 78-120db - *F*_1,20_ = 15.63, *P* = 0.0009; 82-120db - *F*_1,20_ = 14.04, *P* = 0.001; 86-120db - *F*_1,10_ = 14.17, *P* = 0.001; 90-120db - *F*_1,8_ = 14.98, *P* = 0.001). However, the amplitude of the startle response decreased in *Nes*-*Cre*:*Errβ*^*lox*/*lox*^ mice when the intensity of the prepulse tone increased. *Crh* expression was measured in the hindbrain of *Nes*-*Cre*:*Errβ*^*lox*/*lox*^ mice and WT mice. Indeed, *Nes*-*Cre*:*Errβ*^*lox*/*lox*^ mice have decreased expression of *Crh* and *Crhr2* relative to WT (Figure 4b, *F*_1,10_ = 6.54, *P* = 0.03 and Figure 4c, *F*_1,10_ = 6.23, *P* = 0.03). These results indicate alterations in the excitatory pathway that generates a startle response, but not the inhibitory pathway arising from the pedunculopontine tegmental nucleus associated with PPI [41-43]. The increased acoustic startle response in *Nes*-*Cre*:*Errβ*^*lox*/*lox*^ mice may thus arise from altered activity of the excitatory pathway involving *Crh* and *Crhr2* expression and the pontine reticular nucleus, bed nucleus of the stria terminalis, amygdala, and hypothalamus [37-39,43-48]. The hindbrain excitatory pathways, which include catecholaminergic projections to the paraventricular nucleus of the hypothalamus, increase *Crh* expression in the hypothalamus, suggesting that hindbrain signaling may alter the HPA-axis feedback loop [49].

## Discussion

ERRs are involved with energy balance and metabolism [14-18]. Using mice globally deficient for *Errβ*, we have shown that *Errβ* modulates body composition, stress signaling, and hypothalamic neuropeptide expression (Table 2). *Errβ* gene dosage affected body composition and stress response with increased fat mass and corticosterone levels in *Sox2*-*Cre*:*Errβ*^+/*lox*^ mice and decreased fat mass and corticosterone levels in *Sox2*-*Cre*:*Errβ*^*lox*/*lox*^ mice (Table 1 and Figure 2). Additionally, central nervous system-specific *Errβ* deletion alters stress associated with the acoustic startle response pathways (Figure 4).

**Table 2:** **Summary of phenotype difference between *Sox2***-***Cre***:***Errβ***^+/***lox***^, ***Sox2***-***Cre***:***Errβ***^***lox***/***lox***^**and *Nes2***-***Cre***:***Errβ***^***lox***/***lox***^**mice**, **relative to wild type** (**WT**)

**Phenotype**	***Sox2*- *Cre *:** ***Errβ***^+/***lox***^	***Sox2*- *Cre *:** ***Errβ***^***lox***/***lox***^	***Nes2*- *Cre *:** ***Errβ***^***lox***/***lox***^	
Body Composition				
Body weight	↑,	↓,	↑	
Fat mass	↑,	↓,	NC	
Lean mass	↓,	↓,	↑	
Hormone and Neuropeptides				
Corticosterone	↑,	↓,	NA	
Corticosterone (DY131)	↑,	↓,	NA	
*Crh* expression	↑,	↑,	↓	
*Crh* expression (DY131)	↑↑,	↑↑,	NA	
*Npy* expression	↑,	↑,	↓	
*Agrp* expression	↑,	↑,	NA	
Stress Behavior				
Acoustic startle response	NA,	NA,	↑	
Meal patterns				
Total pellets consumed	↑,	↑,	NC	
Inter Meal interval (IMI)	NC,	↓,	↓	
Satiety Ratio	↓^#^,	↓,	↓	

↑ – increase, ↓ – decrease, NC – no change, ↑↑ – increase relative to levels with no DY131 treatment, NA – not available, ^#^*P* = 0.05 relative to WT; *Nes2*-*Cre*:*Errβ*^*lox/lox*^ data adapted from [14].

Hypothalamic expression of *Npy* and *Agrp*, orexigenic factors that increase fat mass and food intake [50-52], increased in both *Sox2*-*Cre*:*Errβ*^+/*lox*^ and *Sox2*-*Cre*:*Errβ*^*lox*/*lox*^ mice (Figure 1). These results suggest that increased anabolic neuropeptide expression may be due to central or peripheral mechanisms that are activated following global deletion of *Errβ*. Increased *Npy* and *Agrp* expression may be due to differences in leptin levels from adipose mass. Increased fat mass and lean mass were measured in *Sox2*-*Cre*:*Errβ*^+/*lox*^ mice, although decreased fat mass and lean mass were measured in highly-active *Sox2*-*Cre*:*Errβ*^*lox*/*lox*^ mice at nine months of age (Table 1). Expression of *Npy* and *leptin* are coordinately regulated, as Npy blunts the effects of leptin and increased leptin levels decrease *Npy* expression [23,53-55]. Thus, *Sox2*-*Cre*:*Errβ*^+/*lox*^ mice may consume more food and increase *Npy* expression and fat mass due to leptin resistance; *Sox2*-*Cre*:*Errβ*^*lox*/*lox*^ mice may increase *Npy* expression to compensate for decreased fat mass arising from increased physical activity (Figure 1 and Table 1). In support of this, *Nes*-*Cre*:*Errβ*^*lox*/*lox*^ mice have increased lean mass, no change in physical activity and have decreased *Npy* expression in the hindbrain [14]. Changes in body composition emerged prior to changes in body weight, suggesting that both peripheral and central signals may be altered to regulate the development of increased fat mass (Table 1).

The opposite phenotypes that are seen in the *Sox2*-*Cre*:*Errβ*^+/*lox*^ and *Sox2*-*Cre*:*Errβ*^*lox*/*lox*^ mice may arise from the ability of Errβ or Errγ to regulate gene transcription as both homodimers and Errβ/Errγ heterodimers [1,6,7,9,10]. Errβ/Errγ heterodimers have been predicted to exist, but to our knowledge it has not been directly detected *in vivo*[1]. RIP140 is a nuclear receptor corepressor that regulates cellular metabolism [56-58]. RIP140 enhanced transcriptional activity for all three mouse *Err* genes [59]. Mice lacking RIP140 are lean, with increased metabolic rate and insulin sensitivity [58]. Similarly, *Sox2*-*Cre*:*Errβ*^*lox*/*lox*^ mice are lean with increased metabolic rate (Table 1 and [14]), and *Nes*-*Cre*:*Errβ*^*lox*/*lox*^ have increased lean mass, increased metabolic rate and insulin sensitivity [14]. Since deletion of both *Errβ* and *RIP140* exhibit similar phenotypes, this suggests that increased lean mass relative to fat mass, metabolic rate and insulin sensitivity may arise from both the RIP140 corepressor and Errβ [59].

ChIP-seq analysis derived from embryonic stem cells revealed that Errβ binds the regulatory element of two genes associated with Crh activity — Corticotropin releasing hormone binding protein (*Crhbp*) and Corticotropin releasing hormone receptor 2 (*Crhr2*) — as well as one gene associated with whole-body energy balance and stress responses, Cholecystokinin B receptor (*Cckbr*) [60]. We hypothesize that *Errβ*, *Crhbp* and *Crhr2* may modulate stress signaling by altering the biological activity of Crh in extrahypothalamic sites and/or corticosterone feedback or secretion [32,39,48,61-64]. Disruption of Errβ-dependent regulation of expression of *Cckbr* and/or *Crhr2* may at least partially explain the abnormal meal patterns and stress behaviors (e.g. acoustic startle response, *Crh* expression or corticosterone levels) observed in *Sox2*-*Cre*:*Errβ*^*lox*/*lox*^, *Sox2*-*Cre*:*Errβ*^+/*lox*^, and *Nes*-*Cre*:*Errβ*^*lox*/*lox*^ mice [14].

Crh is expressed in the paraventricular nucleus of the hypothalamus and initiates ACTH release from the pituitary [40,65]. Crh has since been found to be synthesized in extra-hypothalamic sites, where it also acts to modulate stress response and food intake [40,65-67]. ERR family members also modulate stress responses by regulating glucocorticoid receptor activity in muscle and neuroblastoma cell lines [22,33]. Further, *Errβ* and *Crh* are expressed in similar regions of the hindbrain [29]. Here we demonstrate that *Errβ* deletion modulates corticosterone levels after exposure to restraint stress, with increased levels in *Sox2*-*Cre*:*Errβ*^+/*lox*^ mice and decreased levels in *Sox2*-*Cre*:*Errβ*^*lox*/*lox*^ mice relative to WT (Figure 2). Neural connections projecting to the hypothalamus from extrahypothalamic sites, such as the hindbrain, may also regulate hypothalamic Crh release and *Crh* expression [30,49,68-70].

Biological activity of Crh is inhibited by Crhbp, and Errβ binds to the promoter region of the *Crhbp* gene [60,71], which contains three ERE half sites [72]. Mice that overexpress Crhbp have increased *Crh* expression, potentially resulting from a compensatory response aimed at ameliorating disruptions in stress response [73]. Similarly, increased *Crh* expression was observed when *Errβ* was reduced (*Sox2*-*Cre*:*Errβ*^+/*lox*^) or eliminated (*Sox2*-*Cre*:*Errβ*^+/*lox*^) in somatic tissue, and Errγ was activated using DY131 (Figure 3). Therefore, we propose that partial or complete deletion of *Errβ* may alter *Crh* expression by modulating transcription of *Crhbp* or *Crhr2*, resulting in altered corticosterone secretion. Furthermore, *Sox2*:*Errβ*^*lox*/*lox*^ mice lack corticosterone secretion after restraint stress (Figure 2), which may result from altered *Crhr2* expression (Figure 4c) and changes in negative feedback. Therefore, brain regions that express *Crhr2* may show reduced Crh signaling (Figure 4b and 4c), as in the hindbrain [64].

Errβ binds to *cis*-regulatory regions of the *Cckbr* gene [60], which is expressed in the hindbrain [29,74] and the corresponding gene maps to a genomic locus of the genome associated with obesity [75]. *Cckbr* deficient mice (*Cckbr*^-/-^) display a similar phenotype to *Sox2*-*Cre*:*Errβ*^+/*lox*^ mice, and have increased body weight and food intake, which may arise from changes in Cholecystokinin (Cck) signaling (e.g. satiety), and increased metabolism [74,76]. However, *Cckbr*^-/-^ mice also have blunted stress responses associated with anxiety-like behavior [77] and increased *Npy* expression [78], which resembles the phenotype of *Sox2*-*Cre*:*Errβ*^*lox*/*lox*^ mice (Figure 1 and Table 1). Therefore, heterodimers of Errβ alone, or Errβ in combination with ERRγ, may regulate *Cckbr* transcription, thereby partially accounting for the differences in the phenotypes seen in *Sox2*-*Cre*:*Errβ*^+/*lox*^ and *Sox2*-*Cre*:*Errβ*^*lox*/*lox*^ mice (Table 2). Differences in developmental compensation arising from Errβ and/or Errγ may also contribute to the phenotype differences in *Sox2*-*Cre*:*Errβ*^+/*lox*^*and Sox2*-*Cre*:*Errβ*^*lox*/*lox*^ mice.

*Nes*-*Cre*:*Errβ*^*lox*/*lox*^ mice show increased *Errγ* expression relative to WT animals [14], while mice deficient for *Errγ* show increased *Errβ* expression [17]. This suggests that homozygous mice have reciprocal patterns of *Errβ* and *Errγ* expression, potentially arising from developmental compensation and heterozygous mice may partially lack this compensation, contributing to phenotype differences. The Errβ/Errγ agonist (DY131) increased *Crh* expression more when *Errβ* expression was reduced (*Sox2*:*Errβ*^+/*lox*^ mice) than when *Errβ* expression was absent (*Sox2*:*Errβ*^*lox*/*lox*^ mice) (Figure 3). These results suggest that the ratio of Errβ to Errγ signaling may contribute to the observed difference in *Crh* expression, *Crhr2* expression and corticosterone secretion in the two genotypes examined.

*Sox2*-*Cre*:*Errβ*^*lox*/*lox*^ and *Sox2*-*Cre*:*Errβ*^+/*lox*^ mice have alterations in the HPA axis (Figures 2 and 3). *Npy*, which modulates corticosterone levels [79], is altered in *Sox2*-*Cre*:*Errβ*^+/*lox*^ and *Sox2*-*Cre*:*Errβ*^*lox*/*lox*^ (Figures 1 and 2). Both Crh and Npy have been implicated in modulating the acoustic startle response [32,36,40,80], which is altered in *Nes*-*Cre*:*Errβ*^*lox*/*lox*^ mice (Figure 4 and [14]). Given the results reported here, the phenotype differences between *Sox2*-*Cre*:*Errβ*^+/*lox*^ and *Sox2*-*Cre*:*Errβ*^*lox*/*lox*^ mice may specifically arise from altered *Crh* expression and corticosterone levels as a result of changes in Errβ-dependent regulation of *Crhbp* or *Crhr2* transcription, as well as through interactions of Errβ with Errγ. However, since little is known about Errβ/Errγ heterodimers or how different Err family homo and heterodimers may potentially regulate *Crhbp* or *Crhr2* transcription deserves further investigation.

Our data suggest that central *Errβ* modulates stress responses, food intake and body weight, although it remains to be determined whether peripheral *Errβ* also modulates components of the HPA axis and acoustic startle response. *Nes*-*Cre*:*Errβ*^*lox*/*lox*^ mice lack *Errβ* in the hindbrain and have decreased expression of *Crh*, *Crhr2* and *Npy*[14], suggesting that neuromodulators involved with the acoustic startle response reside in the hindbrain to modulate stress and anxiety. However, other changes in neural circuitry (e.g. altered *Cckbr* expression) regulating the acoustic startle response in *Nes*-*Cre*:*Errβ*^*lox*/*lox*^ mice are likely to exist and remain to be identified.

## Conclusions

Mice heterozygous for *Errβ* deletion have increased fat mass and stress hormone secretion after restraint stress, while those homozygous for *Errβ* deletion have decreased fat mass and secrete higher baseline levels of stress hormones. These effects may be modulated by components of the HPA axis, such as *Crh*, *Crhbp*, *Crhr2*, *Npy or Cckrb*. Central Errβ signaling influences stress associated behavior (e.g. the acoustic startle response), possibly through regulation of *Npy*, *Crh* and *Crhr2* expression in the hindbrain or hypothalamic projections to the amygdala [32,62,63,80]. Since the neural circuitry controlling the acoustic startle response is well-conserved between rodents and humans [36,81], these data suggest that ERRβ or ERRγ may be promising candidates for pharmacological treatment of excessive anxiety or stress levels in humans.

## Methods

### Animals, housing, food intake, and physical activity measurement

*Sox2*-*Cre*:*Errβ*^*lox*/*lox*^, *Sox2*-*Cre*:*Errβ*^+/*lox*^, and wild-type (WT) (*Errβ*^*lox*/*lox*^) mice were generated as previously described [26]. Briefly, Errβ mice have a conditional allele, with loxP sites flanking exon 2 of the *Errβ* gene that encodes the DNA binding domain (exon 2) [26]. Expression of cre recombinase will excise the loxP-flanked exon 2 from the *Errβ* gene. Sox2-Cre deletes *Errβ* from all embryonic tissues and Nestin-Cre deletes *Errβ* from developing neural tissue. *Sox2*-*Cre*:*Errβ*^*lox*/*lox*^ mice completely lack functional *Errβ* because both alleles have been removed. *Sox2*-*Cre*:*Errβ*^+/*lox*^ have one wild-type allele of the *Errβ* gene, since the other allele has been excised by the loxP sites. These two mouse lines enable us to address possible phenotypic differences due to differences in gene dosage. Wild-type (WT) mice used for these studies were homozygous for the floxed Errβ allele. Mice were maintained on a 12:12 hour light–dark cycle in a temperature- and humidity-regulated vivarium and had *ad libitum* access to standard laboratory chow (2018, Harlan-Teklad, Harlan Laboratories, Frederick, MD, USA) and water at all times. Different cohorts of mice were analyzed at three weeks and nine months of age. Food intake data and physical activity levels were collected as previously described [14]. Physical activity levels were measured by detecting and counting horizontal beam breaks in a 40 cm × 40 cm × 30 cm plexiglass chamber (Digiscan, Accuscan Instruments, Columbus, OH). All experimental procedures were performed in accordance with the Johns Hopkins University School of Medicine Institutional Animal Care and Use Committee and the National Institutes of Health *Guide for the Care and Use of Laboratory Animals*.

### In situ *hybridization assay* (*ISH*) *and quantitative real*-*time PCR*

ISH was performed as previously described [14,82]. Briefly, digoxigenin cRNA probes to *Npy* and *agouti*-*related protein* (*Agrp*) were synthesized using the Brain Molecular Anatomy Project (BMAP) library containing sequence-verified expressed sequence tags. BMAP clones were purified using a PureLink plasmid miniprep kit per manufacturer’s protocol (Invitrogen, Carlsbad, CA, USA) and synthesized using a T3 or T7 RNA polymerase (Roche, Indianapolis, IN, USA). The riboprobe was purified using an RNA extraction kit per manufacturer’s protocol (RNeasy, Qiagen, Valencia, CA, USA). Brains were collected from mice, fresh frozen in OCT compound (Tissue Tek, Fisher Scientific, Pittsburgh, PA, USA), and cut using a cryostat into 25-μm sections onto Superfrost Plus slides (Fisher Scientific, Pittsburgh, PA, USA). Hindbrain dissection, mRNA extraction and quantitative real-time PCR was conducted as previously described [14,83]. Briefly, RNA was extracted (RNeasy, Qiagen, Valencia, CA, USA) and cDNA was synthesized using 1 μg of mRNA using Superscript II reverse transcriptase (Invitrogen) and random primers (Invitrogen). Quantitative PCR primer sequences were obtained from PrimerBank and conducted for *Crh*: fwd – 5’ CCTCAGCCGGTTCTGATCC 3’ and rev – 5’ GCGGAAAAAGTTAGCCGCAG 3’, *Crhr2*: fwd – 5’ CATCCACCACGTCCGAGAC 3’ and rev – 5’ CTCGCCAGGATTGACAAAGAA 3’ and *18S* fwd – 5’ GCAATTATTCCCCATGAACG 3’ and rev- 5’ GGCCTCACTAAACCATCCAA 3’. The Ct value generated was normalized to *18S* in order to obtain a ΔCt value, followed by generating the 2^-ΔΔCt^ value by normalizing the data to control animals as previously described [84].

### Restraint stress test, corticosterone radioimmunoassay, and DY131 injections

Baseline blood glucocorticoid levels were measured and mice were placed into a restraining tube (one mouse/tube) for one hour. Upon removal from the restraining tube, blood samples were collected again. Animals were then returned to their housing and blood samples were collected after a one-hour recovery period. Blood was collected in heparin-coated tubes and centrifuged at 3800 rpm for 20 min at 4°C. Corticosterone assays were performed with a radioimmunoassay kit for corticosterone per manufacturer’s directions (MP Biomedicals, Solon, OH, USA). DY131 (Tocris, Bristol, BS11, United Kingdom) at a dose of 10 μM/g body weight was injected, and data for meal patterns collected as previously described [14].

### Prepulse inhibition (PPI) of acoustic startle response

Startle reactivity and PPI were measured using two startle chambers located inside a sound-attenuating chamber (San Diego Instruments, San Diego, CA, USA). Mice were placed in a Plexiglass tube within the soundproof PPI box for a five-minute acclimation period, which provides exposure to a continuous background noise (70 dB) to elicit an increase in startle amplitude [43]. Mice were then exposed for five minutes without any startle stimulus. The PPI session then began and mice were randomly exposed to the following trials: pulse alone (120 dB), no stimulus, or five prepulse combinations (a 20 ms non-startling prepulse at 74, 78, 82, 86, or 90 dB, followed by an 80 ms startle stimulus at 120 dB). The force from the startle reaction was recorded by an accelerometer with SR-LAB software (San Diego Instruments). Results were analyzed by PPI percentage, which was calculated as:

$$\left(\mathrm{mean}\;\mathrm{startle}\;\mathrm{amplitude}\;\mathrm{on}\;\mathrm{pulse}\;\mathrm{alone}\right)-\left(\mathrm{mean}\;\mathrm{startle}\;\mathrm{amplitude}\;\mathrm{on}\;\mathrm{prepulse}\right)/\left(\mathrm{mean}\;\mathrm{startle}\;\mathrm{amplitude}\;\mathrm{on}\;\mathrm{pulse}\;\mathrm{alone}\right).$$

### Statistical analysis

All value comparisons were made using one-way ANOVA to identify individual differences between groups, and P < 0.05 was considered significant (Statistica v.8.0, Tulsa, OK, USA).

## Abbreviations

ACTH: Adrenocorticotropic hormone; Agrp: Agouti-related protein; Crh: Corticotropin-releasing hormone; Crhbp: Corticotropin releasing hormone binding protein; Crhr2: Corticotropin releasing hormone receptor 2; ERR: Estrogen-related receptor; ERRE: Estrogen-related receptor response element; HPA: Hypothalamic-pituitary-adrenal axis; IMI: Inter meal interval; ISH: *In situ* hybridization; Npy: Neuropeptide Y; PPI: Prepulse inhibition; WT: Wild-type.

## Competing interests

The authors declare that they have no competing interests.

## Authors’ contributions

MSB, GWW, and SB; MSB conducted all research; RDS provided technical support for measuring corticosterone levels; MSB analyzed data; MSB drafted the manuscript and MSB, GWW, and SB edited the final version. All authors read and approved the final manuscript.

## References

[B1] Giguere V Transcriptional control of energy homeostasis by the estrogen-related receptors Endocr Rev 2008 13 677 696 10.1210/er.2008-0017 18664618

[B2] Giguere V Yang N Segui P Evans RM Identification of a new class of steroid hormone receptors Nature 1988 13 91 94 10.1038/331091a0 3267207

[B3] Pettersson K Svensson K Mattsson R Carlsson B Ohlsson R Berkenstam A Expression of a novel member of estrogen response element-binding nuclear receptors is restricted to the early stages of chorion formation during mouse embryogenesis Mech Dev 1996 13 211 223 10.1016/0925-4773(95)00479-3 8652414

[B4] Lu D Kiriyama Y Lee KY Giguere V Transcriptional regulation of the estrogen-inducible pS2 breast cancer marker gene by the ERR family of orphan nuclear receptors Cancer Res 2001 13 6755 6761 11559547

[B5] Johnston SD Liu X Zuo F Eisenbraun TL Wiley SR Kraus RJ Mertz JE Estrogen-related receptor alpha 1 functionally binds as a monomer to extended half-site sequences including ones contained within estrogen-response elements Mol Endocrinol 1997 13 342 352 10.1210/me.11.3.342 9058380

[B6] Vanacker JM Pettersson K Gustafsson JA Laudet V Transcriptional targets shared by estrogen receptor- related receptors (ERRs) and estrogen receptor (ER) alpha, but not by ERbeta Embo J 1999 13 4270 4279 10.1093/emboj/18.15.4270 10428965 PMC1171503

[B7] Vanacker JM Bonnelye E Chopin-Delannoy S Delmarre C Cavailles V Laudet V Transcriptional activities of the orphan nuclear receptor ERR alpha (estrogen receptor-related receptor-alpha) Mol Endocrinol 1999 13 764 773 10.1210/me.13.5.764 10319326

[B8] Deblois G Hall JA Perry MC Laganiere J Ghahremani M Park M Hallett M Giguere V Genome-wide identification of direct target genes implicates estrogen-related receptor alpha as a determinant of breast cancer heterogeneity Cancer Res 2009 13 6149 6157 19622763 10.1158/0008-5472.CAN-09-1251

[B9] Huppunen J Aarnisalo P Dimerization modulates the activity of the orphan nuclear receptor ERRgamma Biochem Biophys Res Commun 2004 13 964 970 10.1016/j.bbrc.2003.12.194 14751226

[B10] Gearhart MD Holmbeck SM Evans RM Dyson HJ Wright PE Monomeric complex of human orphan estrogen related receptor-2 with DNA: a pseudo-dimer interface mediates extended half-site recognition J Mol Biol 2003 13 819 832 10.1016/S0022-2836(03)00183-9 12654265

[B11] Giguere V To ERR in the estrogen pathway Trends Endocrinol Metab 2002 13 220 225 10.1016/S1043-2760(02)00592-1 12185669

[B12] Chen F Zhang Q McDonald T Davidoff MJ Bailey W Bai C Liu Q Caskey CT Identification of two hERR2-related novel nuclear receptors utilizing bioinformatics and inverse PCR Gene 1999 13 101 109 10.1016/S0378-1119(98)00619-2 10072763

[B13] Eudy JD Yao S Weston MD Ma-Edmonds M Talmadge CB Cheng JJ Kimberling WJ Sumegi J Isolation of a gene encoding a novel member of the nuclear receptor superfamily from the critical region of Usher syndrome type IIa at 1q41 Genomics 1998 13 382 384 10.1006/geno.1998.5345 9676434

[B14] Byerly MS Al Salayta M Swanson RD Kwon K Peterson JM Wei Z Aja S Moran TH Blackshaw S Wong GW Estrogen-related receptor beta deletion modulates whole-body energy balance via estrogen-related receptor gamma and attenuates neuropeptide Y gene expression Eur J Neurosci 2013 13 1033 1047 10.1111/ejn.12122 23360481 PMC3618562

[B15] Herzog B Cardenas J Hall RK Villena JA Budge PJ Giguere V Granner DK Kralli A Estrogen-related receptor alpha is a repressor of phosphoenolpyruvate carboxykinase gene transcription J Biol Chem 2006 13 99 106 10.1074/jbc.M509276200 16267049

[B16] Luo J Sladek R Bader JA Matthyssen A Rossant J Giguere V Placental abnormalities in mouse embryos lacking the orphan nuclear receptor ERR-beta Nature 1997 13 778 782 10.1038/42022 9285590

[B17] Dufour CR Wilson BJ Huss JM Kelly DP Alaynick WA Downes M Evans RM Blanchette M Giguere V Genome-wide orchestration of cardiac functions by the orphan nuclear receptors ERRalpha and gamma Cell Metab 2007 13 345 356 10.1016/j.cmet.2007.03.007 17488637

[B18] Alaynick WA Kondo RP Xie W He W Dufour CR Downes M Jonker JW Giles W Naviaux RK Giguere V ERRgamma directs and maintains the transition to oxidative metabolism in the postnatal heart Cell Metab 2007 13 13 24 10.1016/j.cmet.2007.06.007 17618853

[B19] Miller DB O'Callaghan JP Neuroendocrine aspects of the response to stress Metabolism 2002 13 5 10 12040534 10.1053/meta.2002.33184

[B20] Mitchell AL Pearce SH Autoimmune Addison disease: pathophysiology and genetic complexity Nat Rev Endocrinol 2012 13 306 316 10.1038/nrendo.2011.245 22290360

[B21] Napier C Pearce SH Autoimmune Addison's disease Presse Med 2012 13 e626 e635 10.1016/j.lpm.2012.09.010 23177474

[B22] Trapp T Holsboer F Nuclear orphan receptor as a repressor of glucocorticoid receptor transcriptional activity J Biol Chem 1996 13 9879 9882 10.1074/jbc.271.17.9879 8626619

[B23] Bjorntorp P Do stress reactions cause abdominal obesity and comorbidities? Obes Rev 2001 13 73 86 10.1046/j.1467-789x.2001.00027.x 12119665

[B24] Erickson JC Ahima RS Hollopeter G Flier JS Palmiter RD Endocrine function of neuropeptide Y knockout mice Regul Pept 1997 13 199 202 10.1016/S0167-0115(97)01007-0 9272634

[B25] Frankish HM Dryden S Hopkins D Wang Q Williams G Neuropeptide Y, the hypothalamus, and diabetes: insights into the central control of metabolism Peptides 1995 13 757 771 10.1016/0196-9781(94)00200-P 7479313

[B26] Chen J Nathans J Estrogen-related receptor beta/NR3B2 controls epithelial cell fate and endolymph production by the stria vascularis Dev Cell 2007 13 325 337 10.1016/j.devcel.2007.07.011 17765677

[B27] Onishi A Peng GH Poth EM Lee DA Chen J Alexis U de Melo J Chen S Blackshaw S The orphan nuclear hormone receptor ERRbeta controls rod photoreceptor survival Proc Natl Acad Sci U S A 2010 13 11579 11584 10.1073/pnas.1000102107 20534447 PMC2895124

[B28] Gofflot F Chartoire N Vasseur L Heikkinen S Dembele D Le Merrer J Auwerx J Systematic gene expression mapping clusters nuclear receptors according to their function in the brain Cell 2007 13 405 418 10.1016/j.cell.2007.09.012 17956739

[B29] Lein ES Hawrylycz MJ Ao N Ayres M Bensinger A Bernard A Boe AF Boguski MS Brockway KS Byrnes EJ Genome-wide atlas of gene expression in the adult mouse brain Nature 2007 13 168 176 10.1038/nature05453 17151600

[B30] Grill HJ Hayes MR Hindbrain neurons as an essential hub in the neuroanatomically distributed control of energy balance Cell Metab 2012 13 296 309 10.1016/j.cmet.2012.06.015 22902836 PMC4862653

[B31] Gray TS Cassell MD Kiss JZ Distribution of pro-opiomelanocortin-derived peptides and enkephalins in the rat central nucleus of the amygdala Brain Res 1984 13 354 358 10.1016/0006-8993(84)90386-X 6087978

[B32] Yang FC Connor J Patel A Doat MM Romero MT Neural transplants. effects On startle responses in neonatally MSG-treated rats Physiol Behav 2000 13 333 344 10.1016/S0031-9384(99)00256-5 10869600

[B33] Wang SC Myers S Dooms C Capon R Muscat GE An ERRbeta/gamma agonist modulates GRalpha expression, and glucocorticoid responsive gene expression in skeletal muscle cells Mol Cell Endocrinol 2010 13 146 152 10.1016/j.mce.2009.07.012 19631715

[B34] Yu DD Forman BM Identification of an agonist ligand for estrogen-related receptors ERRbeta/gamma Bioorg Med Chem Lett 2005 13 1311 1313 10.1016/j.bmcl.2005.01.025 15713377

[B35] Plappert CF Pilz PK The acoustic startle response as an effective model for elucidating the effect of genes on the neural mechanism of behavior in mice Behav Brain Res 2001 13 183 188 10.1016/S0166-4328(01)00299-6 11682109

[B36] Koch M The neurobiology of startle Prog Neurobiol 1999 13 107 128 10.1016/S0301-0082(98)00098-7 10463792

[B37] Lee Y Lopez DE Meloni EG Davis M A primary acoustic startle pathway: obligatory role of cochlear root neurons and the nucleus reticularis pontis caudalis J Neurosci 1996 13 3775 3789 8642420 10.1523/JNEUROSCI.16-11-03775.1996 PMC6578836

[B38] Davis M Falls WA Campeau S Kim M Fear-potentiated startle: a neural and pharmacological analysis Behav Brain Res 1993 13 175 198 10.1016/0166-4328(93)90102-V 8136044

[B39] Inglefield JR Schwarzkopf SB Kellogg CK Alterations in behavioral responses to stressors following excitotoxin lesions of dorsomedial hypothalamic regions Brain Res 1994 13 151 161 10.1016/0006-8993(94)91534-2 8137151

[B40] Liang KC Melia KR Campeau S Falls WA Miserendino MJ Davis M Lesions of the central nucleus of the amygdala, but not the paraventricular nucleus of the hypothalamus, block the excitatory effects of corticotropin-releasing factor on the acoustic startle reflex J Neurosci 1992 13 2313 2320 1607942 10.1523/JNEUROSCI.12-06-02313.1992 PMC6575925

[B41] Plappert CF Pilz PK Schnitzler HU Factors governing prepulse inhibition and prepulse facilitation of the acoustic startle response in mice Behav Brain Res 2004 13 403 412 10.1016/j.bbr.2003.10.025 15196809

[B42] Hoffman HS Ison JR Reflex modification in the domain of startle: I. Some empirical findings and their implications for how the nervous system processes sensory input Psychol Rev 1980 13 175 189 7375610

[B43] Leumann L Sterchi D Vollenweider F Ludewig K Fruh H A neural network approach to the acoustic startle reflex and prepulse inhibition Brain Res Bull 2001 13 101 110 10.1016/S0361-9230(01)00607-4 11704346

[B44] Yeomans JS Frankland PW The acoustic startle reflex: neurons and connections Brain Res Brain Res Rev 1995 13 301 314 10.1016/0165-0173(96)00004-5 8806018

[B45] Heilig M Koob GF Ekman R Britton KT Corticotropin-releasing factor and neuropeptide Y: role in emotional integration Trends Neurosci 1994 13 80 85 10.1016/0166-2236(94)90079-5 7512773

[B46] Fendt M Koch M Schnitzler HU NMDA receptors in the pontine brainstem are necessary for fear potentiation of the startle response Eur J Pharmacol 1996 13 1 6 10.1016/S0014-2999(96)00749-2 9007504

[B47] Alon T Zhou L Perez CA Garfield AS Friedman JM Heisler LK Transgenic mice expressing green fluorescent protein under the control of the corticotropin-releasing hormone promoter Endocrinology 2009 13 5626 5632 10.1210/en.2009-0881 19854866 PMC2795705

[B48] Meloni EG Gerety LP Knoll AT Cohen BM Carlezon WA Jr Behavioral and anatomical interactions between dopamine and corticotropin-releasing factor in the rat J Neurosci 2006 13 3855 3863 10.1523/JNEUROSCI.4957-05.2006 16597740 PMC6674129

[B49] Khan AM Kaminski KL Sanchez-Watts G Ponzio TA Kuzmiski JB Bains JS Watts AG MAP kinases couple hindbrain-derived catecholamine signals to hypothalamic adrenocortical control mechanisms during glycemia-related challenges J Neurosci 2011 13 18479 18491 10.1523/JNEUROSCI.4785-11.2011 22171049 PMC3293627

[B50] Patel HR Qi Y Hawkins EJ Hileman SM Elmquist JK Imai Y Ahima RS Neuropeptide Y deficiency attenuates responses to fasting and high-fat diet in obesity-prone mice Diabetes 2006 13 3091 3098 10.2337/db05-0624 17065347

[B51] Segal-Lieberman G Trombly DJ Juthani V Wang X Maratos-Flier E NPY ablation in C57BL/6 mice leads to mild obesity and to an impaired refeeding response to fasting Am J Physiol Endocrinol Metab 2003 13 E1131 E1139 12582011 10.1152/ajpendo.00491.2002

[B52] Wortley KE Anderson KD Yasenchak J Murphy A Valenzuela D Diano S Yancopoulos GD Wiegand SJ Sleeman MW Agouti-related protein-deficient mice display an age-related lean phenotype Cell Metab 2005 13 421 427 10.1016/j.cmet.2005.11.004 16330327

[B53] Sainsbury A Cusin I Doyle P Rohner-Jeanrenaud F Jeanrenaud B Intracerebroventricular administration of neuropeptide Y to normal rats increases obese gene expression in white adipose tissue Diabetologia 1996 13 353 356 10.1007/BF00418353 8721783

[B54] Schwartz MW Baskin DG Bukowski TR Kuijper JL Foster D Lasser G Prunkard DE Porte D Jr Woods SC Seeley RJ Specificity of leptin action on elevated blood glucose levels and hypothalamic neuropeptide Y gene expression in ob/ob mice Diabetes 1996 13 531 535 10.2337/diab.45.4.531 8603777

[B55] Schwartz MW Seeley RJ Campfield LA Burn P Baskin DG Identification of targets of leptin action in rat hypothalamus J Clin Invest 1996 13 1101 1106 10.1172/JCI118891 8787671 PMC507530

[B56] Christian M White R Parker MG Metabolic regulation by the nuclear receptor corepressor RIP140 Trends Endocrinol Metab 2006 13 243 250 10.1016/j.tem.2006.06.008 16815031

[B57] Rosell M Jones MC Parker MG Role of nuclear receptor corepressor RIP140 in metabolic syndrome Biochim Biophys Acta 2011 13 919 928 10.1016/j.bbadis.2010.12.016 21193034 PMC3117993

[B58] Leonardsson G Steel JH Christian M Pocock V Milligan S Bell J So PW Medina-Gomez G Vidal-Puig A White R Nuclear receptor corepressor RIP140 regulates fat accumulation Proc Natl Acad Sci USA 2004 13 8437 8442 10.1073/pnas.0401013101 15155905 PMC420412

[B59] Castet A Herledan A Bonnet S Jalaguier S Vanacker JM Cavailles V Receptor-interacting protein 140 differentially regulates estrogen receptor-related receptor transactivation depending on target genes Mol Endocrinol 2006 13 1035 1047 10.1210/me.2005-0227 16439465

[B60] Chen X Xu H Yuan P Fang F Huss M Vega VB Wong E Orlov YL Zhang W Jiang J Integration of external signaling pathways with the core transcriptional network in embryonic stem cells Cell 2008 13 1106 1117 10.1016/j.cell.2008.04.043 18555785

[B61] Meloni EG Reedy CL Cohen BM Carlezon WA Jr Activation of raphe efferents to the medial prefrontal cortex by corticotropin-releasing factor: correlation with anxiety-like behavior Biol Psychiatry 2008 13 832 839 10.1016/j.biopsych.2007.10.016 18061145 PMC2362385

[B62] Lyons AM Thiele TE Neuropeptide Y conjugated to saporin alters anxiety-like behavior when injected into the central nucleus of the amygdala or basomedial hypothalamus in BALB/cJ mice Peptides 2010 13 2193 2199 10.1016/j.peptides.2010.09.009 20863864 PMC2971693

[B63] Deo GS Dandekar MP Upadhya MA Kokare DM Subhedar NK Neuropeptide Y Y1 receptors in the central nucleus of amygdala mediate the anxiolytic-like effect of allopregnanolone in mice: Behavioral and immunocytochemical evidences Brain Res 2010 13 77 86 20059983 10.1016/j.brainres.2009.12.088

[B64] Chalmers DT Lovenberg TW De Souza EB Localization of novel corticotropin-releasing factor receptor (CRF2) mRNA expression to specific subcortical nuclei in rat brain: comparison with CRF1 receptor mRNA expression J Neurosci 1995 13 6340 6350 7472399 10.1523/JNEUROSCI.15-10-06340.1995 PMC6577987

[B65] Grill HJ Markison S Ginsberg A Kaplan JM Long-term effects on feeding and body weight after stimulation of forebrain or hindbrain CRH receptors with urocortin Brain Res 2000 13 19 28 10.1016/S0006-8993(00)02193-4 10837794

[B66] Bledsoe AC Oliver KM Scholl JL Forster GL Anxiety states induced by post-weaning social isolation are mediated by CRF receptors in the dorsal raphe nucleus Brain Res Bull 2011 13 117 122 10.1016/j.brainresbull.2011.03.003 21396988 PMC3109218

[B67] Hammack SE Pepin JL DesMarteau JS Watkins LR Maier SF Low doses of corticotropin-releasing hormone injected into the dorsal raphe nucleus block the behavioral consequences of uncontrollable stress Behav Brain Res 2003 13 55 64 10.1016/S0166-4328(03)00133-5 14659570

[B68] Grill HJ Kaplan JM The neuroanatomical axis for control of energy balance Front Neuroendocrinol 2002 13 2 40 10.1006/frne.2001.0224 11906202

[B69] Grill HJ Distributed neural control of energy balance: contributions from hindbrain and hypothalamus Obesity (Silver Spring) 2006 13 Suppl 5 216S 221S 17021370 10.1038/oby.2006.312

[B70] Sawchenko PE Swanson LW Central noradrenergic pathways for the integration of hypothalamic neuroendocrine and autonomic responses Science 1981 13 685 687 10.1126/science.7292008 7292008

[B71] Potter E Behan DP Linton EA Lowry PJ Sawchenko PE Vale WW The central distribution of a corticotropin-releasing factor (CRF)-binding protein predicts multiple sites and modes of interaction with CRF Proc Natl Acad Sci U S A 1992 13 4192 4196 10.1073/pnas.89.9.4192 1315056 PMC525659

[B72] Behan DP Potter E Lewis KA Jenkins NA Copeland N Lowry PJ Vale WW Cloning and structure of the human corticotrophin releasing factor-binding protein gene (CRHBP) Genomics 1993 13 63 68 10.1006/geno.1993.1141 8198617

[B73] Burrows HL Nakajima M Lesh JS Goosens KA Samuelson LC Inui A Camper SA Seasholtz AF Excess corticotropin releasing hormone-binding protein in the hypothalamic-pituitary-adrenal axis in transgenic mice J Clin Invest 1998 13 1439 1447 10.1172/JCI1963 9525987 PMC508722

[B74] Clerc P Coll Constans MG Lulka H Broussaud S Guigne C Leung-Theung-Long S Perrin C Knauf C Carpene C Penicaud L Involvement of cholecystokinin 2 receptor in food intake regulation: hyperphagia and increased fat deposition in cholecystokinin 2 receptor-deficient mice Endocrinology 2007 13 1039 1049 17122076 10.1210/en.2006-1064

[B75] Samuelson LC Isakoff MS Lacourse KA Localization of the murine cholecystokinin A and B receptor genes Mamm Genome 1995 13 242 246 10.1007/BF00352408 7613026

[B76] Miyasaka K Ichikawa M Ohta M Kanai S Yoshida Y Masuda M Nagata A Matsui T Noda T Takiguchi S Energy metabolism and turnover are increased in mice lacking the cholecystokinin-B receptor J Nutr 2002 13 739 741 11925470 10.1093/jn/132.4.739

[B77] Horinouchi Y Akiyoshi J Nagata A Matsushita H Tsutsumi T Isogawa K Noda T Nagayama H Reduced anxious behavior in mice lacking the CCK2 receptor gene Eur Neuropsychopharmacol 2004 13 157 161 10.1016/S0924-977X(03)00103-2 15013032

[B78] Chen H Kent S Morris MJ Is the CCK2 receptor essential for normal regulation of body weight and adiposity? Eur J Neurosci 2006 13 1427 1433 10.1111/j.1460-9568.2006.05016.x 16965546

[B79] Leibowitz SF Sladek C Spencer L Tempel D Neuropeptide Y, epinephrine and norepinephrine in the paraventricular nucleus: stimulation of feeding and the release of corticosterone, vasopressin and glucose Brain Res Bull 1988 13 905 912 10.1016/0361-9230(88)90025-1 3224284

[B80] Gutman AR Yang Y Ressler KJ Davis M The role of neuropeptide Y in the expression and extinction of fear-potentiated startle J Neurosci 2008 13 12682 12690 10.1523/JNEUROSCI.2305-08.2008 19036961 PMC2621075

[B81] CaH Landis WA The Startle Pattern 1939 New York: Farrar and Rinehart

[B82] Blackshaw S Snyder SH Developmental expression pattern of phototransduction components in mammalian pineal implies a light-sensing function J Neurosci 1997 13 8074 8082 9334383 10.1523/JNEUROSCI.17-21-08074.1997 PMC6573733

[B83] Byerly MS Simon J Lebihan-Duval E Duclos MJ Cogburn LA Porter TE Effects of BDNF, T3, and corticosterone on expression of the hypothalamic obesity gene network in vivo and in vitro Am J Physiol Regul Integr Comp Physiol 2009 13 R1180 R1189 10.1152/ajpregu.90813.2008 19158410 PMC2698606

[B84] Livak KJ Schmittgen TD Analysis of relative gene expression data using real-time quantitative PCR and the 2(-Delta Delta C(T)) Method Methods 2001 13 402 408 10.1006/meth.2001.1262 11846609

